# Significant Reduction of Chenodeoxycholic Acid and Glycochenodeoxycholic Acid in the Elderly with Severe COVID-19

**DOI:** 10.3390/biom15070943

**Published:** 2025-06-28

**Authors:** Shiyang Liu, Wen Xu, Bo Tu, Zhiqing Xiao, Xue Li, Lei Huang, Xin Yuan, Shengdong Luo, Juanjuan Zhou, Xinxin Yang, Junlian Yang, De Chang, Weiwei Chen, Fu-Sheng Wang

**Affiliations:** 1 Chinese People’s Liberation Army (PLA) Medical School, Beijing 100853, China; 18811509502@163.com; 2 Senior Department of Infectious Diseases, The Fifth Medical Center of Chinese PLA General Hospital, National Clinical Research Center for Infectious Diseases, Beijing 100073, China; xuwen302yy@163.com (W.X.); tub_302@163.com (B.T.); lixue7796@163.com (X.L.); huangleiwa@sina.com (L.H.); xinyuannatile@sina.com (X.Y.); lsdwork2010@163.com (S.L.); zhoujuanjuan_302@163.com (J.Z.); yxinxin88@163.com (X.Y.); tianshi427@foxmail.com (J.Y.); 3 Department of Pulmonary and Critical Care Medicine, The Seventh Medical Center, College of Pulmonary and Critical Care Medicine of The Eighth Medical Center, Chinese PLA General Hospital, Beijing 100007, China; xiao_zq2025@163.com (Z.X.); changde@301hospital.com.cn (D.C.); 4 Yu-Yue Pathology Scientific Research Center, Chongqing 401329, China

**Keywords:** CDCA, GCDCA, elderly COVID-19 patients, disease progression, inflammatory response

## Abstract

Elderly individuals infected with SARS-CoV-2 are at higher risk of developing cytokine storms and severe outcomes, yet specific biomarkers remain unclear. In this study, we investigated the alteration of primary bile acid metabolism in elderly patients with severe COVID-19 using untargeted metabolomics (*n* = 31), followed by targeted metabolomics to compare patients with disease progression (*n* = 16) to those without (*n* = 48). Significant reductions in chenodeoxycholic acid (CDCA) and glycochenodeoxycholic acid (GCDCA) levels were identified in severe cases, with GCDCA levels at admission correlating strongly with peak inflammatory markers. In vitro, CDCA, GCDCA, and their receptors, Farnesoid X Receptor (FXR) and Takeda G-protein-coupled receptor 5 (TGR5), effectively inhibited the inflammatory response induced by SARS-CoV-2. NOD-like receptor pathway, activated by SARS-CoV-2, may modulate inflammatory cytokines under the treatment of CDCA, GCDCA, and TGR5. CDCA and GCDCA levels at admission predicted disease progression, suggesting their potential as biomarkers for severe COVID-19 in the elderly and highlighting their regulatory role in inflammation, pointing to new therapeutic avenues.

## 1. Introduction

COVID-19 was a pandemic globally from December 2019 to May 2023 [1], resulting in approximately 765 million infections and 6.92 million deaths [2]. The elderly [3], those with underlying diseases [4], and those who have not been vaccinated [5] are at high risk for SARS-CoV-2 infection and clinical deterioration. Among them, elderly individuals are more likely to have combined risk factors of elder age and comorbidities. The severe illness rate, intensive care unit (ICU) admission rate, and mortality rate after SARS-CoV-2 infection are significantly higher in the elderly than in other adults and children [6,7,8]. Therefore, the research on the characteristics of severe illness in older COVID-19 patients has certain clinical significance.

Previous reports suggested that bile acids and their receptors were involved in the pathogenesis of COVID-19. Specifically, bile acids can prevent viral entry by interfering with the binding of the spike protein to angiotensin-converting enzyme II (ACE2), thereby inhibiting viral uptake and replication [9]. Additionally, they modulate immune responses indirectly. For example, the secondary bile acid UDCA reduces ACE2 expression by inhibiting the farnesoid X receptor (FXR), which may lower susceptibility to SARS-CoV-2 infection [10]. Meanwhile, cytokine storm is considered the main driver of COVID-19 disease progression [11]. FXR activation attenuates inflammatory responses by downregulating IL-6 expression and secretion in Caco-2 cells infected with SARS-CoV-2 via suppression of the NF-κB signaling pathway [12]. The levels of glycocholic acid (GCA), taurocholic acid (TCA), taurochenodeoxycholic acid (TCDCA), and glycochenodeoxycholic acid (GCDCA) in the plasma of moderate COVID-19 patients are significantly lower than those in mild patients [13]. Based on these findings, bile acid and its receptors indeed play a complex role in SARS-CoV-2 infection and immune regulation. However, whether bile acids can serve as biomarkers of severe COVID-19 disease in clinics is still unclear, and the relationship between bile acids and cytokine storm remains to be further studied.

This study primarily focused on the disease progression of elderly patients with COVID-19 to find the predictive biomarkers and unveil the underlying mechanism of them in inflammatory regulation. By analyzing the expression characteristics of primary bile acids in the peripheral blood, we identified CDCA and GCDCA as candidate biomarkers for the disease progression of COVID-19 in the elderly, and they suppressed inflammatory responses induced by SARS-CoV-2 infection in vitro. These results suggested that disturbed primary bile acids metabolism was a key characteristic of older COVID-19 patients, which might be the potential therapeutic target to prevent disease progression in the elderly.

## 2. Materials and Methods

### 2.1. Patient Inclusion and Sample Collection

This study enrolled patients aged over 60 hospitalized for COVID-19 at the Fifth and the Eighth Medical Center of the PLA General Hospital from November 2022 to April 2023. COVID-19 diagnosis was confirmed via RT-PCR or antigen tests alongside epidemiological and clinical data. Exclusion criteria included progressive tumors, immune diseases, immunosuppressant use, liver disorders, organ transplantation, and non-fasting blood sampling. Patients were classified into Non-severe (mild/moderate) and Severe (severe/critical) groups based on China’s clinical guidelines [14].

We conducted Study 1 and Study 2 by untargeted metabolomics and targeted metabolomics, respectively (Supplementary Figure S1). Study 1 collected plasma from 8 severe (Severe group) and 23 non-severe patients (Non-severe group) from November 2022 to December 2023; Study 2 enrolled 64 non-severe patients by April 2023, categorized into severe progression (NS-S, 16 cases) and non-severe progression (NS-N, 48 cases) groups. Baseline data included demographics, comorbidities, vaccination, symptoms, treatment, and immune-related parameters. Immune-related parameters comprised immune cell counts and proportions (leukocyte, lymphocytes, neutrophils, monocytes), procalcitonin (PCT), C-reactive protein (CRP), interleukin-6 (IL-6), as well as neutrophil-to-lymphocyte ratio (NLR), platelet-to-lymphocyte ratio (PLR), lymphocyte-to-monocyte ratio (LMR), and systemic immune–inflammation index (SII) [15,16,17]. Blood samples were collected within 24 h of admission, processed into serum or plasma, and stored at −80 °C for detection.

### 2.2. Cell Culture, Viral Stimulation, and Bile Acid Intervention

The THP-1 cell line was obtained from the National Platform for Sharing Experimental Cell Resources of China. It was cultivated in RPMI-1640 medium (Gibco, Grand Island, CA, USA) containing 10% FBS (Gibco, Grand Island, CA, USA), 1% streptomycin–penicillin (Gibco, Grand Island, CA, USA) and 55 nM β-mercaptoethanol (Solarbio, Beijing, China).

The SARS-CoV-2 (XBB.1.16) was kindly gifted by Professor Wei Liu’s laboratory. The virus was inactivated by water bath at 56 °C for 30 min [18,19,20] in the biosafety level 3 (BSL3) facility in the State Key Laboratory of Pathogen and Biosecurity, Beijing Institute of Microbiology and Epidemiology. THP-1 cells were inoculated at a density of 8×105/mL in 12-well plates and cultivated in medium with (MOI = 1) or without virus. After virus stimulation, the medium with or without CDCA (MCE, Monmouth Junction, NJ, USA), GCDCA (MCE, Monmouth Junction, NJ, USA), GW4064 (FXR receptor agonist; MCE, Monmouth Junction, NJ, USA), and INT-777 (Takeda G-protein-coupled receptor 5 (TGR5) agonist; MCE, Monmouth Junction, NJ, USA) was added, and the cells were incubated at 37 °C (5% CO_2_) for 24 h. Guggulsterone (FXR antagonist; MCE, Monmouth Junction, NJ, USA) and SBI-115 (TGR5 antagonist; MCE, Monmouth Junction, NJ, USA) were used to block FXR and TGR5, respectively. After 24 h, supernatants were collected and stored at −80 °C. Five replicates were set up for each group, and the results were obtained from at least three independent experiments.

### 2.3. LC-MS/MS Untargeted Metabolomics

A total of 100 μL of plasma was mixed with 400 μL of methanol–acetonitrile (2:1, containing 2 μg/mL L-2-chlorophenylalanine; Thermo Fisher Scientific, Waltham, MA, USA), vortexed, ultrasonicated in an ice-water bath, and stored at −40 °C. After centrifugation, 200 μL of supernatant was dried, reconstituted in methanol–water (1:4; Thermo Fisher Scientific, Waltham, MA, USA), and filtered through a 0.22 μm organic phase filter. Analysis was performed using an ACQUITY UPLC I-Class plus system (Waters, Milford, MA, USA) coupled with a QE high-resolution mass spectrometer (Thermo Fisher Scientific, Waltham, MA, USA). Metabolites were identified using HMDB, Lipidmaps, Metlin, and a self-built database, with bioinformatics analysis conducted using R packages (v3.6.1).

### 2.4. LC-MS/MS Targeted Bile Acids Metabolomics

A total of 100 μL of serum was mixed with 200 μL of methanol–acetonitrile (2:1, containing isotope internal standards and 1 mM BHT), vortexed, ultrasonicated, and centrifuged. The supernatant was diluted, filtered, and analyzed using a Nexera UHPLC LC-30A (Shimadzu, Kyoto, Japan) system coupled with an AB Sciex Qtrap 5500 mass spectrometer (AB SCIEX, Redwood, CA, USA) in MRM mode. Metabolite quantification was performed using SCIEX OS-MQ software (https://sciex.com/products/software/, accessed on 28 June 2024), with absolute concentrations calculated based on standard curves and dilution factors.

### 2.5. qPCR (Quantitative Real-Time PCR)

Total RNA was extracted from THP-1 cells with the EasyPure^®^ Fast Cell RNA Kit (TransGen Biotech Co., Beijing, China). Reverse transcription was conducted using PrimeScript™ RT Master Mix (TAKARA, Kyoto, Japan). TB Green^®^ Premix Ex Taq™ II (TAKARA, Kyoto, Japan) was utilized for qPCR reactions. Data acquisition and analysis were performed using Design & Analysis 2.8.0 (Thermo Fisher Scientific, Waltham, MA, USA) software. The inflammatory genes examined were FXR, TGR5, IL-1β, IL-6, TNF-α, IL-8, and CXCL5 (Supplementary Table S1).

### 2.6. Cytokine Quantification

Cytokine levels (IL-1β, IL-6, TNF-α, IL-8) were assessed using QBPlex^®^ Multiple Immunoassays for Flow (Quantobio, Beijing, China). CXCL5 was examined by ELISA for (Multi Sciences, Hangzhou, Zhejiang, China).

### 2.7. RNA Sequencing of THP-1 Cells

Library preparation is performed using the Optimal Dual-mode mRNA Library Prep Kit (BGI-Shenzhen, Shenzhen, Guangdong, China). The raw sequencing data were filtered using SOAPnuke (v1.5.6). Subsequent data analysis, visualization, and mining were performed using the Dr. Tom multi-omics data analysis system (https://biosys.bgi.com (accessed on 18 December 2024)).

### 2.8. Statistical Analysis

Normally distributed continuous data were expressed as mean ± s.d. and analyzed using Student’s *t*-test or ANOVA, while non-normally distributed data were expressed as median (IQR) and analyzed using Mann–Whitney or Kruskal–Wallis tests. Pairwise comparisons were performed using Tukey’s test. Count data were analyzed by chi-square tests, and multivariate binary logistic regression was used for risk factor analysis. Data with >25% missing values or patients with >25% missing bile acid data were excluded. Values below the lower limit of detection (LLOD) or upper limit of detection (ULOD) were replaced with half LLOD or ULOD, respectively. ROC curves were used for progression prediction, and Spearman rank correlation was applied for correlation analysis. A two-sided *p* < 0.05 was considered significant. Analyses were performed using PRISM (v8) and SPSS (v27), with replicates detailed in figure legends.

## 3. Results

### 3.1. Alterations of Primary Bile Acids Biosynthesis in Peripheral Blood of Elderly Patients with Severe COVID-19

**Figure 1:**
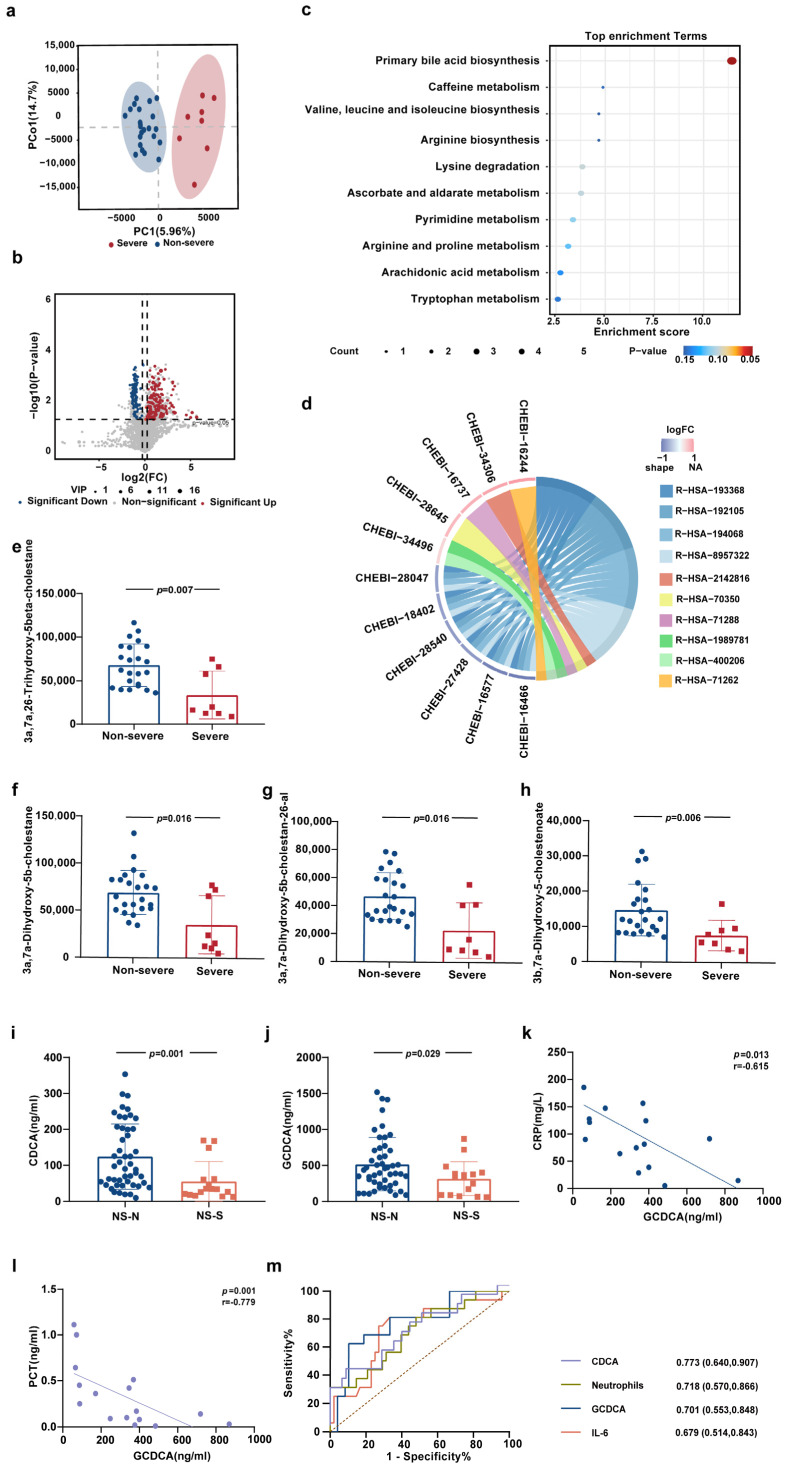
The biosynthesis of primary bile acids in the peripheral blood of elderly patients with severe COVID-19 altered significantly. (**a**) PCA of metabolites in peripheral blood of elderly patients with (Severe group, *n* = 8) or without (Non-severe group, *n* = 23) severe COVID-19 at admission. (**b**) The volcano plot presented the upregulated and downregulated metabolites in the Severe group vs. the Non-severe group. Screening criteria for differential metabolites: VIP > 1, *p* < 0.05, |log2FC| > 1.2. (**c**) Bubble chart of enriched pathways in metabolism by KEGG analysis of differential metabolites between the Severe group and Non-severe group. Terms are labeled and sorted by enrichment score. (**d**) Chord Diagram of enriched pathways by Reactome analysis of differential metabolites between the Severe group and Non-severe group. The same color series (blue series and green series) represented a subordinate relationship among the pathways. (**e**–**h**) Comparison of four intermediate metabolites of cholesterol metabolism to CDCA detected by untargeted metabolomics in the Severe group and Non-severe group at admission. (**i**,**j**) Comparison of CDCA and GCDCA detected by targeted metabolomics in the NS-S group and NS-N group at admission. (**k**,**l**) Correlation analysis of GCDCA in peripheral blood of NS-S patients with CRP and PCT at admission. (**m**) ROC curves of CDCA, GCDCA, IL-6, and neutrophils (%) at admission in predicting severe progression of COVID-19 during hospitalization. Univariate statistical analysis was performed using Student’s *t*-test (for normally distributed data) or Mann–Whitney U test (for non-parametric data). The resulting *p* values were adjusted for multiple testing via the Benjamini–Hochberg false discovery rate (FDR) correction, with q < 0.05 considered statistically significant. (**b**,**e**–**j**) Correlation analysis was conducted using Spearman rank correlation (**k**–**m**).

In order to understand the metabolic characteristics of peripheral blood in elderly patients with severe COVID-19, the untargeted metabolomics detection was conducted in 8 severe patients and 23 non-severe patients (Supplementary Table S2). The principal component analysis (PCA) indicated a distinct separation of metabolites between the Severe group and the Non-severe group, suggesting differences in metabolite expression between the two groups (Figure 1a). Compared with the Non-severe group, there were 152 upregulated differential metabolites and 103 downregulated differential metabolites in the Severe group (Figure 1b).

Kyoto Encyclopedia of Genes and Genomes (KEGG) enrichment analysis demonstrated that the biosynthesis of primary bile acids had the highest enrichment score in the module of the metabolic pathway (Figure 1c). The intermediate metabolites of cholesterol metabolism to CDCA in this pathway, namely 3a, 7a, 26-Trihydroxy-5beta-cholestane, 3a, 7a-Dihydroxy-5b-cholestane, and 3a, 7a-Dihydroxy-5b-cholestan-26-al, were all significantly downregulated in the Severe group (Figure 1e–h, Supplementary Figure S2). The top three pathways in the Reactome enrichment analysis were the synthesis of bile acids and bile salts via 7a-hydroxycholesterol, the synthesis of bile acids and bile salts, and the metabolism of bile acids and bile salts (Figure 1d, Supplementary Table S3). 7a-hydroxycholesterol is generated from cholesterol through the rate-limiting enzyme CYP7A1 (the first enzyme in the classical pathway of bile acid synthesis) and then further catalyzed by CYP8B1 for the oxidation and cleavage of the side chain to form CA and CDCA [21]. The results of targeted metabolomics detection also confirmed that CDCA was significantly downregulated in the Severe group (Supplementary Figure S3a). The above results suggested that the low expression of primary bile acid CDCA in peripheral blood is a crucial characteristic of elderly patients with severe COVID-19.

### 3.2. CDCA and GCDCA Were Significantly Downregulated in Elderly Patients with Severe COVID-19

Through untargeted metabolomics analysis, we identified downregulation of primary bile acid biosynthesis, especially CDCA, in elderly patients with severe COVID-19; however, its potential as a biomarker for disease severity remains unclear. Thus, we enrolled a total of 64 elderly COVID-19 patients who were non-severe at admission to detect the relationship between their primary bile acids concentrations and disease progression. During hospitalization, 16 cases progressed to severe disease (NS-S group), and 48 cases did not (NS-N group).

The basic information, clinical features, and immune indicators at baseline of the NS-N group and NS-S group are shown in Table 1. There was no significant difference between the two groups in terms of sex, underlying comorbidities, and lifestyle; however, the age of the NS-S group was significantly higher than that of the NS-N group (*p* < 0.05), consistent with previous knowledge that aging is a risk factor for severity. There were no significant differences between the two groups in terms of vaccination, the time from the onset to sample collection, the time of positive nucleic acid, and the onset symptoms, yet the length of hospital stay of the NS-S group was significantly longer than that of the NS-N group (*p* < 0.05), which indicate patients in the NS-S group need more treatment. Regarding immune-related indicators, the percentage of neutrophils in the NS-S group was significantly elevated, while the percentage and count of lymphocytes were significantly reduced, and the levels of PCT, IL-6, and NLR were significantly increased (*p* < 0.05); those results were in line with previous reports [22,23].

Targeted metabolomics detection revealed that the expression levels of primary bile acids, CDCA and GCDCA, in the peripheral blood of the NS-S group were significantly lower than those in the NS-N group (*p* < 0.05) (Figure 1i,j). The levels of CDCA in the Severe group and the NS-S group were both significantly lower than that in the NS-N group (*p* < 0.05) (Supplementary Figure S3a), suggesting that elderly patients presented the feature of low CDCA expression even before fully meeting the clinical indications for severe COVID-19. The decrease in CDCA expression in the NS-S group was not influenced by the time from disease onset to sample collection and age (Supplementary Figure S3b,c). Furthermore, the expression of GCDCA at admission in the NS-S group was significantly negatively correlated with the peak values of inflammatory markers CRP and PCT during hospitalization (Figure 1k,l), indicating that GCDCA represents a promising molecular target for regulating inflammatory processes. Consequently, both CDCA and GCDCA have the potential to indicate the progression of severe COVID-19 in elderly patients.

### 3.3. CDCA and GCDCA Inhibit the Inflammatory Response Induced by SARS-CoV-2 in THP-1 Cells

Building upon the aforementioned clinical research findings, we hypothesize that CDCA and GCDCA may serve as potential therapeutic targets for attenuating the SARS-CoV-2-induced inflammatory cascade and associated cytokine storm. Meanwhile, it is noted that the significant increase in monocytes producing IL-6 in severe COVID-19 patients suggests that monocytes are key contributors to cytokine storm in COVID-19 [24]. Furthermore, a clinical research has shown that 6% monocytes could be infected by SARS-CoV-2 [25]. Modifications in monocyte functionality and activation states are also recognized as a potential mechanism underlying increased morbidity and mortality due to SARS-CoV-2 infection in older adults [26]. Thus, we established a SARS-CoV-2-stimulated THP-1 cell model to elucidate whether CDCA and GCDCA regulated inflammatory responses in monocytes induced by SARS-CoV-2.

**Figure 2:**
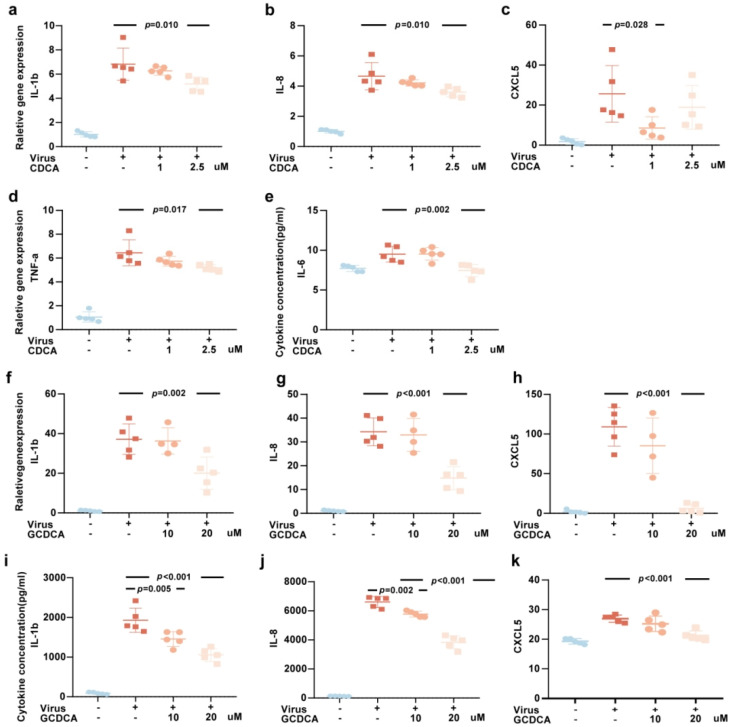
CDCA and GCDCA inhibit the inflammatory response induced by SARS-CoV-2 in THP-1 cells. (**a**–**e**) SARS-CoV-2-stimulated THP-1 cells were treated with 1 μM or 2.5 μM CDCA for 24 h. The intracellular mRNA expression levels of IL-1β (**a**), IL-8 (**b**), CXCL5 (**c**), and TNF-α (**d**) were measured by RT–qPCR. The supernatant protein concentration of IL-6 (**e**) was measured by CBA. These samples derived from the same experiment. (**f**–**k**) SARS-CoV-2-stimulated THP-1 cells were treated with 10 μM or 20 μM GCDCA for 24 h. The intracellular mRNA expression levels of IL-1β (**f**), IL-8 (**g**), and CXCL5 (**h**) were measured by RT–qPCR. The supernatant protein concentrations of IL-1β (**i**) and IL-8 (**j**) were measured by CBA, and CXCL5 (**k**) level was measured by ELISA. These samples derived from the same experiment. Multiple comparisons were performed except for the blank control; *n*  =  5 biologically independent samples. Data are presented as mean  ±  s.d. The two-sided *p* values were examined using one-way ANOVA followed by Tukey’s multiple-comparison test for comparison of continuous variables among multiple groups (**a**–**k**).

Our results revealed that CDCA significantly attenuated the upregulation of IL-1β, IL-8, CXCL5, and TNF-α expression (Figure 2a–d), as well as the increased secretion of IL-6 in THP-1 cells stimulated by SARS-CoV-2 for 24 h (Figure 2e). Additionally, CDCA exhibited a trend toward inhibiting the secretion of IL-1β, IL-8, and CXCL5 (Supplementary Figure S4a–c). Similarly, GCDCA markedly reduced the upregulation of IL-1β, IL-8, and CXCL5 expression (Figure 2f–h), as well as the elevated secretion of IL-1β, IL-8, and CXCL5 (Figure 2i–k), in a concentration-dependent manner following SARS-CoV-2 stimulation. GCDCA also showed a tendency to suppress the secretion of IL-6 and IL-1α (Supplementary Figure S4d,e). These findings suggested that CDCA and GCDCA, at appropriate concentrations, can effectively modulate the inflammatory response triggered by SARS-CoV-2.

### 3.4. The Activation of Bile Acid Receptors FXR and TGR5 Regulate the Inflammatory Response Induced by SARS-CoV-2

FXR and TGR5 serve as the natural endogenous nuclear receptors and membrane receptors for CDCA and GCDCA, respectively. These receptors are ubiquitously expressed in various human cells and tissues, including monocytes [27,28]. To elucidate the roles of FXR and TGR5 in SARS-CoV-2-induced inflammatory responses, we performed a 24 h time-course experiment.

**Figure 3:**
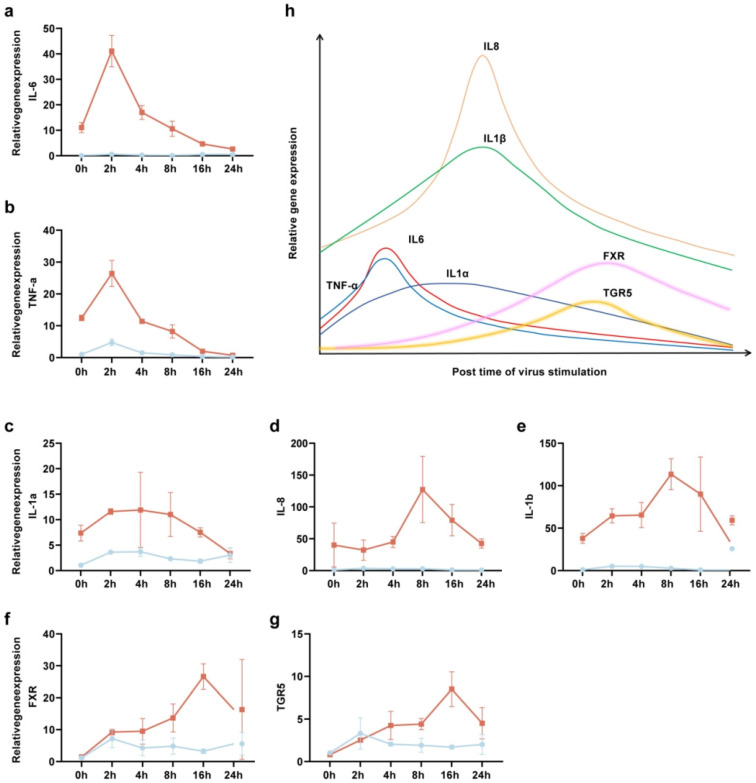
The temporal trends in the mRNA expression levels of FXR, TGR5, and inflammatory cytokines in THP-1 cells stimulated by SARS-CoV-2 over a 0–24 h period. (**a**–**g**) SARS-CoV-2-stimulated THP-1 cells for 0 h, 2 h, 4 h, 8 h, 16 h, and 24  h. The intracellular mRNA expression levels of IL-6 (**a**), TNF-α (**b**), IL-1α (**c**), IL-8 (**d**), IL-1β (**e**), FXR (**f**), and TGR5 (**g**) were measured by RT–qPCR. (**h**) A schematic presentation of the temporal trends in the mRNA expression levels of FXR, TGR5, and inflammatory cytokines in THP-1 cells stimulated by SARS-CoV-2 over a 0–24 h period. These samples derived from the same experiment; *n*  =  5 biologically independent samples. Data are presented as mean  ±  s.d. The orange trajectory represents THP-1 cells stimulatied by SARS-CoV-2, while the blue trajectory depicts unstimulated controls (**a**–**g**).

Our findings revealed that SARS-CoV-2 stimulation of THP-1 cells markedly upregulated the expression of key inflammatory cytokines, such as IL-1α, IL-1β, IL-6, IL-8, and TNF-α (Figure 3a–e). Notably, the peak expression of FXR and TGR5 displayed a temporal delay relative to the peak expression of inflammatory cytokines (Figure 3f,g). Furthermore, the elevation of FXR and TGR5 levels coincided with a subsequent reduction in the expression of inflammatory cytokines (Figure 3h). However, whether the activation of FXR and TGR5 directly contributes to the downregulation of inflammatory cytokines remains to be further investigated.

**Figure 4:**
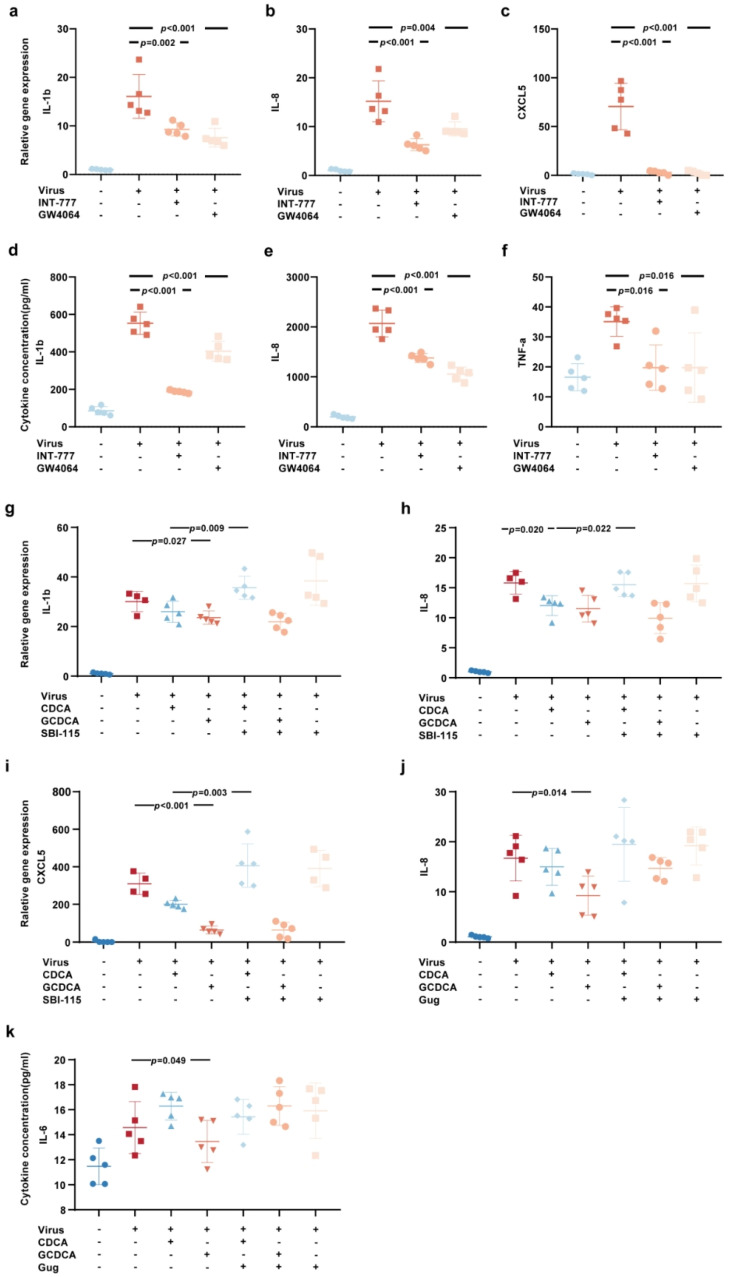
FXR and TGR5 modulate the inflammatory response induced by SARS-CoV-2 in THP-1 cells. (**a**–**f**) THP-1 cells were stimulated by SARS-CoV-2 and treated with 20 μM GW4064 or INT-777 for 24 h. The intracellular mRNA expression levels of IL-1β (**a**), IL-8 (**b**), and CXCL5 (**c**) were measured by RT–qPCR. The supernatant protein concentrations of IL-1β (**d**), IL-8 (**e**), and TNF-α (**f**) were measured by CBA. These samples derived from the same experiment. (**g**–**i**) A total of 3 μM SBI-115 was administered 2 h prior to the 24 h treatment with CDCA and GCDCA to block TGR5 signaling in the context of SARS-CoV-2 stimulation. The intracellular mRNA expression levels of IL-1β (**g**), IL-8 (**h**), and CXCL5 (**i**) were measured by RT–qPCR. These samples derived from the same experiment. (**j**,**k**) A total of 5 μM Guggulsterone was administered 2 h prior to the 24 h treatment with CDCA and GCDCA to block FXR signaling in the context of SARS-CoV-2 stimulation. The intracellular mRNA expression level of IL-8 (**j**) was measured by RT–qPCR. The supernatant protein concentration of IL-6 (**k**) was measured by CBA. These samples derived from the same experiment. Multiple comparisons were performed except for the blank control (**a**–**f**); *n*  =  5 biologically independent samples. Multiple comparisons were performed among virus, V+CDCA/GCDCA, and V+CDCA/GCDCA+antagonist (**g**–**k**), respectively; *n* = 5 biologically independent samples. Data are presented as mean ± s.d. The two-sided *p* values were examined using one-way ANOVA followed by Tukey’s multiple-comparison test for comparison of continuous variables among multiple groups (**a**–**k**).

GW4064 and INT-777, which are specific agonists for FXR and TGR5, respectively. Treatment with GW4064 and INT-777 effectively reversed the upregulation of IL-1β, IL-8, and CXCL5 (Figure 4a–c) and reduced the secretion of IL-1β, IL-8, and TNF-α (Figure 4d–f) induced by SARS-CoV-2. Furthermore, the TGR5 antagonist SBI-115 attenuated the suppressive effect of CDCA on the SARS-CoV-2-induced upregulation of IL-1β and IL-8 (Figure 4g,h) and reversed the inhibitory effect of CDCA on CXCL5 (Figure 4i) secretion. Similarly, the FXR antagonist Guggulsterone (Gug) diminished the inhibitory effects of CDCA and GCDCA on the SARS-CoV-2-induced upregulation of IL-8 expression and reversed the suppression of IL-6 secretion by GCDCA (Figure 4j,k). Collectively, these findings indicate that the activation of FXR or TGR5 mediate anti-inflammatory effects, which are similar to those of CDCA and GCDCA in SARS-CoV-2-induced inflammatory responses. Importantly, the inhibition of either FXR or TGR5 partially attenuates the anti-inflammatory actions of CDCA and GCDCA.

### 3.5. CDCA/GCDCA Might Suppress SARS-CoV-2-Induced Inflammatory Responses by Inhibiting the NOD-like Receptor Signaling Pathway

**Figure 5:**
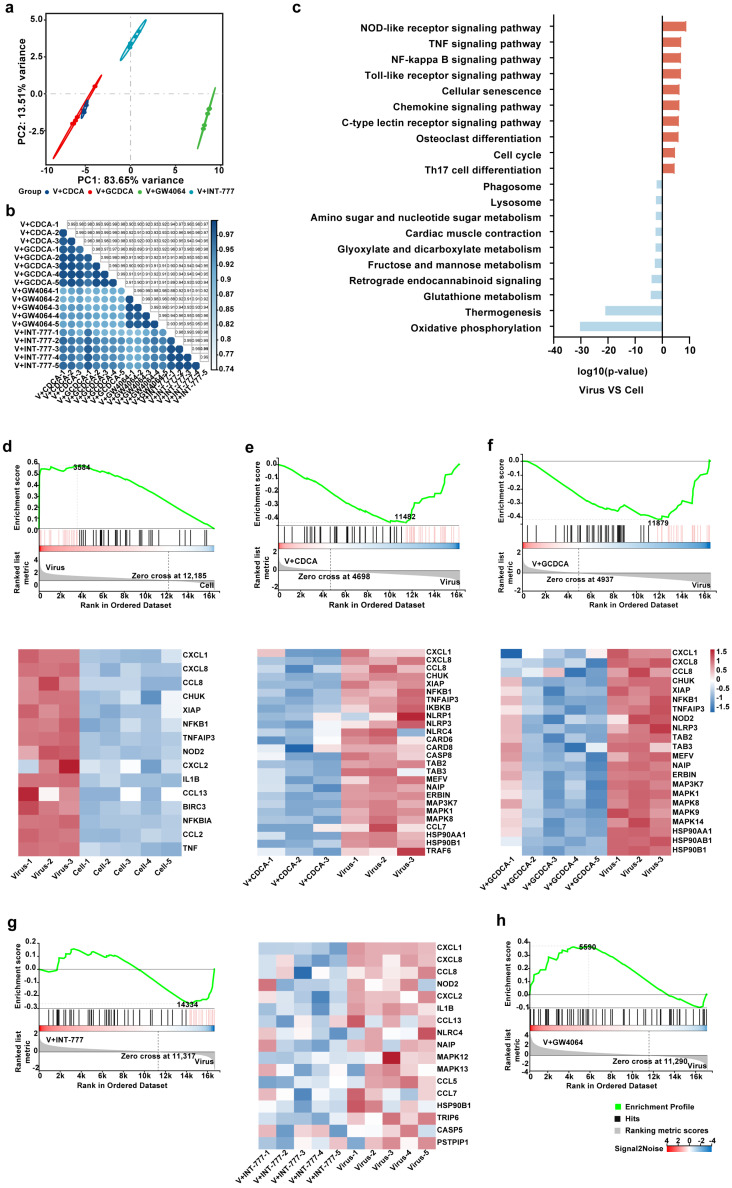
The treatment of CDCA, GCDCA, and TGR5 agonist INT-777 exhibit the mechanistic parallels in modulating the inflammatory response induced by SARS-CoV-2 in THP-1 cells through the NOD-like receptor signaling pathway. (**a**,**b**) RNA sequencing of THP-1 cell line stimulated by SARS-CoV-2 treated with 2.5 μM CDCA, 20 μM GCDCA, 20 μM GW4064, or 20 μM INT-777 for 24 h. PCA (**a**) and sample correlation analysis (**b**) of DEGs among the treatment of four compounds. (**c**) KEGG enrichment of TOP 10 upregulated and downregulated pathways induced by SARS-CoV-2 stimulation. (**d**–**h**) GSEA of NOD-like receptor signaling pathway in THP-1 cells stimulated by SARS-CoV-2 (**e**) and treated with CDCA (**f**), GCDCA (**g**), INT-777 (**h**), and GW4064 (**d**). The enrichment plot has three components. (Top) Enrichment score profile showing the maximum deviation from zero. The green curve represents the running enrichment score. (Middle) The bars indicate gene set member positions in the ranked gene list, while the core enrichment genes are highlighted in red. (Bottom) Red/blue gradient indicates high/low expression of leading-edge genes within the pathway. Gray area plot displays Signal2Noise values across all genes (ranked left-to-right). The heatmap plot indicates variations of the Transcript per Kilobase per Million mapped reads (TPM) between each sample of the two groups.

To investigate the mechanism by which CDCA and GCDCA exert their inhibitory effects on SARS-CoV-2-induced inflammatory responses in monocytes, the transcriptomic profiles of THP-1 cells treated with these bile acids and their receptors were analyzed. The principal component analysis (PCA) (Figure 5a) demonstrated that, in the context of virus stimulation, the differentially expressed genes (DEGs) of the CDCA treatment group were highly concordant with that of the GCDCA treatment group, and the DEGs of the TGR5 agonist (INT-777) treatment group exhibited greater similarity to those of the CDCA and GCDCA treatment groups, respectively. On the other hand, the DEGs of the FXR agonist (GW4064) treatment group had less similarity with the CDCA or GCDCA treatment group. Consistent with this, sample correlation analysis revealed stronger correlations between the INT-777 treatment group and the CDCA/GCDCA treatment groups than between the GW4064 treatment group and the CDCA/GCDCA treatment groups (Figure 5b). These results were coincident with the previous notion that TGR5 preferentially binds to primary bile acids, compared with FXR. These findings suggest that CDCA and GCDCA may exert their effects through a mechanism more closely aligned with TGR5 activation than FXR activation.

Furthermore, enrichment analysis of DEGs identified significant upregulation of multiple inflammation-related signaling pathways, such as the nucleotide-binding oligomerization domain (NOD)-like receptor signaling pathway, TNF signaling pathway, NF-κB signaling pathway, and Toll-like receptor signaling pathway (Figure 5c). Notably, the NOD-like receptor signaling pathway emerged as the most prominently upregulated pathway, highlighting its potential central role in SARS-CoV-2-induced inflammatory responses.

**Figure 6:**
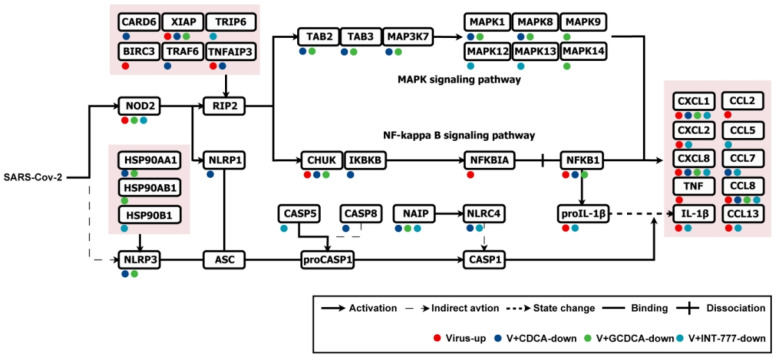
A schematic presentation of the treatment of CDCA, GCDCA, and INT-777 modulating the inflammatory response induced by SARS-CoV-2 in THP-1 cells through the NOD-like receptor signaling pathway.

The modulation of the NOD-like receptor signaling pathway by the four compounds further underscores the mechanistic parallels among the treatment with CDCA, GCDCA, and INT-777. Gene Set Enrichment Analysis (GSEA) revealed that SARS-CoV-2 upregulates the NOD-like receptor signaling pathway by enhancing the expression of leading-edge subset, including CXCL1, CXCL8, C-C motif chemokine ligand 8 (CCL8), X-linked inhibitor of apoptosis protein (XIAP), NFKB subunit 1 (NFKB1), TNF alpha-induced protein 3 (TNFAIP3), NOD-containing protein 2 (NOD2), CXCL2, IL1B, CCL13, baculoviral IAP repeat-containing protein 3 (BIRC3), nuclear factor of kappa light polypeptide gene enhancer in B-cells inhibitor alpha (NFKBIA), CCL2, and TNF (Figure 5e,i). In contrast, treatment with CDCA, GCDCA, and INT-777 resulted in the downregulation of this pathway (Figure 5f–h), whereas treatment with GW4064 did not (Figure 5d). These observations suggest that CDCA and GCDCA seemed to mediate their anti-inflammatory effects against SARS-CoV-2 by modulating the NOD-like receptor signaling pathway through TGR5. To further dissect the mechanistic details of the NOD-like receptor signaling pathway, we performed an in-depth analysis of the leading-edge subsets enriched under SARS-CoV-2 stimulation and following treatment with CDCA, GCDCA, and INT-777 (Figure 6). Our findings indicate that SARS-CoV-2 activates the NF-κB signaling pathway via the NOD2-receptor-interacting serine/threonine-protein kinase 2 (RIP2) axis, thereby driving the secretion of pro-inflammatory cytokines such as TNF, IL-1β, CXCL1, CXCL8. Notably, CDCA, GCDCA, and INT-777 suppressed the expression of NOD2 or upstream genes of RIP2, effectively inhibiting the pro-inflammatory actions of the NF-κB signaling pathway. Furthermore, these compounds attenuated inflammatory cytokine released by targeting the NOD2-RIP2 axis to inhibit the MAPK signaling pathway and by downregulating NLR family pyrin domain containing 3 (NLRP3) and NLRP1, which subsequently reduced the activation of Caspase 1 (CASP1). Collectively, these results highlight the pivotal role of the NOD-like receptor signaling pathway in mediating the anti-inflammatory effects of CDCA, GCDCA, and TGR5 activation in the context of SARS-CoV-2-induced inflammation.

### 3.6. CDCA and GCDCA Predict the Progression of Severe COVID-19 in Elderly Patients

Our in vitro experiments demonstrated the anti-inflammatory effects of CDCA, GCDCA, and their receptors. To further assess the potential of CDCA and GCDCA as biomarkers for predicting severe COVID-19 progression, we evaluated the relationship between their expression levels at admission and the clinical outcomes in elderly COVID-19 patients.

A univariate logistic regression on the factors that exhibited significant differences between the NS-S group and NS-N groups was performed. Subsequently, we conducted a multivariate logistic regression on the factors that demonstrated statistical significance after the univariate logistic regression. The results indicated that an increased percentage of neutrophils (%) (OR [95% CI] = 1.082 [1.014, 1.155], *p* = 0.018), elevated IL-6 (OR [95% CI] = 1.026 [1.003, 1.050], *p* = 0.028), and decreased CDCA (OR [95% CI] = 0.978 [0.960, 0.996], *p* = 0.018) at admission were independent risk factors for the severe progression of COVID-19 in elderly patients (Table 2).

To further analyze the predictive value of CDCA and GCDCA on the severe progression of COVID-19, we conducted ROC analysis on the predictive value of CDCA, GCDCA, neutrophils, and IL-6 (Figure 1m). The results revealed that CDCA had a superior predictive capacity, followed by neutrophils (%). CDCA demonstrated better predictive performance (AUC = 0.773, 95% CI: 0.640–0.907) than GCDCA (AUC = 0.701, 95% CI: 0.553–0.848). The optimal cutoff values were determined as 36.77 ng/mL for CDCA and 418.96 ng/mL for GCDCA. Overall, these findings highlight the potential of decreased levels of CDCA and GCDCA as prognostic biomarkers for the severe progression of COVID-19 in elderly patients.

## 4. Discussion

Advanced age represents a significant risk factor for severe disease progression and mortality in COVID-19 patients [3,6]. It is valuable to depict the pathogenesis of severe older patients infected by SARS-CoV-2 and find new biomarkers to predict the disease severity of those patients. In this study, we identified significant alterations in the primary bile acid biosynthesis pathway in elderly patients with severe COVID-19. Specifically, we observed decreased levels of CDCA and GCDCA at admission in the Non-severe to Severe (NS-S) group. Notably, GCDCA levels at admission demonstrated a significant negative correlation with peak inflammatory markers during hospitalization. Through in vitro experiments, we confirmed the anti-inflammatory effects of CDCA and GCDCA, as well as their respective receptors (FXR and TGR5). We also explored the potential mechanisms underlying these effects, which appear to involve the suppression of the NOD-like receptor signaling pathway. Importantly, our findings reveal that alterations in CDCA and GCDCA levels precede the clinical manifestation of COVID-19 progression to severe states in elderly patients, suggesting their potential utility as early predictive biomarkers.

Differential metabolites identified in COVID-19 patients, especially in severe cases, serve as valuable biomarkers for disease pathophysiology and potential targets for diagnosis, therapy, and immunomodulation [29,30,31]. Our investigation employed a comprehensive two-phase metabolomics approach to identify and validate potential biomarkers in elderly COVID-19 patients with severe manifestations. Initially, we systematically analyzed the metabolic profiles of elderly patients with severe COVID-19 through a cross-sectional study, revealing a significant downregulation of the primary bile acid biosynthesis pathway in this group. Previous studies have reported that several primary bile acids, including GCDCA, and secondary bile acids in the plasma of moderate COVID-19 patients are significantly lower than those in mild patients [13]. Extending these observations, Porru et al. reported significantly lower serum concentrations of GCDCA in non-survivors compared to survivors [32]. Moreover, their observation of elevated GCDCA-3S levels in non-survivors may suggest that enzymes involved in GCDCA-3S metabolism may play a crucial role in COVID-19 pathogenesis. Together, these results support that COVID-19 infection leads to significant perturbations in bile acid metabolism in more serious patients. Building on these consistent findings, we further screened two metabolites, CDCA and GCDCA, as potential peripheral blood biomarkers for indicating progression to severe COVID-19 using a rigorously defined research cohort, exclusively enrolling patients who presented with non-severe COVID-19 at admission. Collectively, we elucidated the predictive value of these biomarkers in the disease progression towards severe COVID-19. Notably, although our study had a relatively small sample size, analyses from a two-phase metabolomics approach consistently indicated that primary bile acid was altered in elderly patients with severe COVID-19. Unlike previous metabolomic studies where reported COVID-19 biomarkers demonstrated limited reproducibility [33], our findings robustly validate the changes in primary bile acid levels in elderly patients with severe COVID-19.

Our research also suggested that bile acid levels correlated with the inflammatory state of COVID-19 patients. The cytokine storm triggered by SARS-CoV-2 infection, characterized by an overwhelming inflammatory response, has been identified as a primary contributor to severe disease progression and mortality in COVID-19 patients [11]. In this study, our results showed that the level of GCDCA at admission demonstrated significant correlations with the peak PCT and CRP levels during hospitalization. This correlation has not been widely reported in studies of COVID-19 infections. Xie et al. reported that patients infected with Severe Fever with Thrombocytopenia Syndrome Virus (SFTSV) exhibit a high GCDCA serum concentration that was negatively correlated with IL-1β and IL-6 expression in serum [34]. The convergence of these observations suggests that the diminished expression of GCDCA may serve as a harbinger for the onset of systemic inflammation and the dysregulated immune response characteristic of severe disease states.

This study also showed that bile acids could regulate inflammatory responses of monocytes induced by SARS-CoV-2. Our in vitro experiments validated the regulatory effects of CDCA, GCDCA, and their receptor (FXR and TGR5) agonists/antagonists on the expression and secretion of inflammatory cytokines, such as IL-1β, IL-8, and CXCL5. Previous studies have reported that GCDCA treatment suppresses the release of IL-6 and TNF-α through the TGR5 receptor in THP-1 cells infected with SFTSV [34]. TGR5 activation inhibits influenza virus-induced IL-1β and CXCL1 production from bone marrow-derived macrophages [13]. FXR activation inhibits SARS-CoV-2-induced IL-6 expression in HEK293 cells and Caco-2 cells [12]. In coincidence with those results, our data supported the anti-inflammation effects of CDCA, GCDCA, FXR activation, and TGR5 activation in THP-1 cells stimulated by SARS-CoV-2. Otherwise, we found that GCDCA exhibited a more evident inhibitory effect on inflammation response than CDCA. In humans, CDCA is predominantly conjugated with glycine to form GCDCA and rarely with taurine to form TCDCA [35]. The introduction of glycine increases amphiphilicity and optimizes the spatial conformation of the molecule, making it easier to interact with the cell membrane [36]. These characteristics of GCDCA may contribute to its ability to better bind to receptors and exert anti-inflammatory effects.

Our findings showed that the NOD-like receptor signaling pathway was one of the most important pathways mediating the anti-inflammatory effects of bile acids and bile acid receptors. Indeed, CDCA, GCDCA, FXR activation, and TGR5 activation have been shown to be involved in multiple inflammatory signal transduction. FXR ligands exert anti-inflammatory activities by antagonizing other signaling pathways, in part through the interaction with other transcription factors, including AP-1, STAT3, NF-κB, TNFα, IL-1, IL-6, cyclooxygenase-1, and cyclooxygenase-2 [37,38]. TGR5 ligands trigger anti-inflammation by regulating NF-κB signaling [39], mTOR/oxidative phosphorylation signaling [40], and cAMP/PKA signaling [41]. Previous investigations have shown that secondary bile acids, particularly UDCA, have immunomodulatory effects on SARS-CoV-2 infection [10]. By activating TGR5, UDCA suppresses immune cell activation and inflammation, suggesting therapeutic potential for mitigating cytokine storm in severe COVID-19 [42]. This study showed that CDCA, GCDCA, and TGR5 exhibited parallel regulatory mechanisms within the NOD-like receptor signaling pathway. NOD2, NLRP3, and NLRP1 were identified as key regulatory targets of these three compounds. The regulatory role of TGR5 on NLRP3 has been well-established in various pathological conditions, including cerebral hemorrhage [41], steatohepatitis [43], and endotoxin-induced inflammatory responses [44]. Our findings highlight that this mechanism also plays a significant role in SARS-CoV-2-induced inflammatory responses.

We have a few limitations of the study. First, our study focused on the specific population of elderly patients, for whom we established stringent inclusion and exclusion criteria, resulting in a limited sample size; therefore, future large-scale and multi-center studies should validate our findings to assess generalizability and robustness. Second, pre-admission medication and vaccination status were not systematically collected, and unmeasured confounders (e.g., genetic/environmental factors) limited our assessment of their effects on disease progression and biomarkers. Future studies should include these factors to clarify their roles. Furthermore, our study provides a link between CDCA, GCDCA, and inflammation response induced by SARS-CoV-2; however, as our study was conducted during the Omicron BA.5.2/BF.7-dominant period in China, further comparative studies with other SARS-CoV-2 variants or respiratory viral infections are needed to determine whether this link represents broader pathogenic mechanisms. Finally, we explored a potential molecular mechanism linking TGR5 to the NOD-like receptor pathway. The role of gut microbiota in them warrants further investigation to inform personalized treatment strategies.

## 5. Conclusions

In summary, our study focused on elderly COVID-19 patients, a vulnerable subgroup, and identified reduced levels of circulating CDCA and GCDCA as predictive biomarkers for severe disease progression. Our exploratory research also suggests that CDCA, GCDCA, and INT-777 inhibit SARS-CoV-2-induced inflammation by suppressing the NOD-like receptor signaling pathway. These findings provide potential biomarkers and therapeutic targets for detecting and treating elderly individuals with severe COVID-19.

## Figures and Tables

**Table 1:** Comparison of personal information, clinical characteristics, and immune indicators at baseline between the NS-N and NS-S groups of elder COVID-19 patients.

	NS-N Group (*n* = 48)	NS-S Group (*n* = 16)	*p*-Value
**Personal information**			
Gender			
Male	30 (62.50%)	14 (87.50%)	0.119
Female	18 (37.50%)	2 (12.50%)	
Age (year) *	76.80 ± 10.93	83.49 ± 10.70	0.037
Underlying comorbidities			
Circulatory system diseases	29 (60.42%)	12 (75.00%)	0.292
Endocrine system diseases	21 (43.75%)	6 (37.50%)	0.661
Digestive system diseases	11 (22.92%)	2 (12.50%)	0.590
Nervous system diseases	12 (25.00%)	5 (31.25%)	0.870
Urinary system diseases	7 (14.58%)	3 (18.75%)	1.000
Respiratory system diseases	5 (10.42%)	0 (0.00%)	0.420
Musculoskeletal system	5 (10.42%)	3 (18.75%)	0.663
Others	5 (10.42%)	0 (0.00%)	0.420
Living habits			
Allergies	4 (8.33%)	1 (6.25%)	1.000
Smoking	3 (6.25%)	0 (0.00%)	0.733
Drinking	4 (8.33%)	1 (6.25%)	1.000
**Clinical information**			
Vaccination			
Unvaccinated	12 (25.00%)	4 (25.00%)	0.986
Vaccinated	17 (35.42%)	6 (37.50%)	
Unclear	19 (39.58%)	6 (37.50%)	
Time from the onset to sample collection (day)	5.50 (3.00, 8.50)	6.00 (2.00, 8.50)	0.749
Time of positive nucleic acid (day)	5.00 (3.00, 8.00)	10.00 (4.00, 15.00)	0.092
Length of hospital stay (day) ***	7.00 (5.00, 10.00)	16.00 (12.00, 20.50)	<0.001
Onset symptoms			
Fever	39 (81.25%)	12 (75.00%)	0.858
Cough	35 (72.92%)	13 (81.25%)	0.739
Expectoration	28 (58.33%)	10 (62.50%)	0.769
Sore throat	15 (31.25%)	4 (25.00%)	0.874
Others	15 (31.25%)	4 (25.00%)	0.874
**Immune-related parameters**			
Leukocyte (10^9^/L)	4.23 (3.61, 6.58)	5.30 (3.69, 6.50)	0.603
Neutrophils (%) *	67.06 ± 14.99	78.49 ± 15.27	0.011
Lymphocytes (%) **	22.42 ± 12.00	12.69 ± 9.78	0.005
Monocytes (%)	8.60 (5.55, 12.65)	6.05 (3.00, 8.70)	0.061
Neutrophils (10^9^/L)	2.73 (2.07, 5.03)	4.18 (3.42, 4.93)	0.195
Lymphocytes (10^9^/L) *	1.07 ± 0.61	0.66 ± 0.49	0.018
Monocytes (10^9^)/L)	0.45 (0.30, 0.56)	0.30 (0.12, 0.53)	0.239
PCT (ng/mL) *	0.05 (0.03, 0.09)	0.12 (0.06, 0.44)	0.032
CRP (mg/L)	20.90 (7.00, 45.25)	30.20 (10.20, 62.60)	0.522
IL-6 (pg/mL) *	13.91 (5.05, 39.67)	32.03 (13.23, 123.80)	0.034
NLR *	2.95 (1.88, 6.87)	5.22 (3.83, 7.84)	0.038
PLR	151.27 (122.81, 258.50)	168.83 (98.40, 317.46)	0.762
LMR	2.16 (1.72, 3.85)	2.27 (0.61, 2.90)	0.351
SII	415.66 (282.17, 1167.54)	645.50 (465.05, 1924.34)	0.225

Note: * means *p*-value < 0.05; ** means *p*-value < 0.01; *** means *p*-value < 0.001. Circulatory system diseases included hypertension, coronary heart disease, arrhythmias, peripheral artery disease, and stroke. Endocrine system diseases included diabetes, gout, and hyperuricemia. Digestive system diseases included gastroesophageal reflux disease and peptic ulcers. Nervous system diseases included Alzheimer’s disease, Parkinson’s disease, epilepsy, multiple sclerosis, peripheral neuropathy, stroke, migraines, stun, neurogenic deafness, depression, insomnia, and anxiety. Urinary system diseases included chronic kidney disease, kidney stones, urinary tract infections, and hyperplasia of the prostate. Respiratory system diseases included chronic obstructive pulmonary disease, asthma, interstitial pneumonia, and pulmonary nodules. Musculoskeletal system diseases included osteoarthritis, osteoporosis, muscular dystrophy, lumbar disc herniation, paralysis, and fractures. Others included pressure sores, cataracts, glaucoma, and eczema.

**Table 2:** Analysis of influencing factors for the severe progression of COVID-19 in elder patients.

Variants	Univariate Logistic Regression		Multivariate Logistic Regression	
	*p*-Value	OR (95%CI)	*p*-Value	OR (95%CI)
Age (years) (60–74)				
75–90	0.610	1.437 (0.357, 5.795)		
>90	0.121	3.067 (0.745, 12.625)		
Neutrophils (%)	0.016	1.054 (1.010, 1.100)	0.018	1.082 (1.014, 1.155)
Lymphocytes (%)	0.009	0.922 (0.868, 0.980)		
Lymphocytes (10^9^/L)	0.021	0.208 (0.055, 0.785)		
PCT (ng/mL)	0.182	3.682 (0.543, 24.991)		
IL-6 (pg/mL)	0.033	3.889 (1.119, 13.516)	0.028	1.026 (1.003, 1.050)
CDCA (ng/mL)	0.015	0.986 (0.975, 0.997)	0.018	0.978 (0.960, 0.996)
GCDCA (ng/mL)	0.058	0.998 (0.995, 1.000)		

Note: PCT, procalcitonin.

## Data Availability

The untargeted (REQ20250214208655) and targeted (REQ20250214208656) metabolomic data presented in the study will be openly available at MetaboLights on 14 February 2026. The RNA sequencing data are openly available at NCBI (BioProject ID: PRJNA1235325).

## Supplementary Materials

The following supporting information can be downloaded at https://www.mdpi.com/article/10.3390/biom15070943/s1, Figure S1: Timeline of patient enrollment, sampling, and detection process; Figure S2: Differential metabolites on the primary bile acid biosynthesis pathway; Figure S3: The expression of CDCA detected by targeted metabolomics in the peripheral blood of elderly COVID-19 patients at admission; Figure S4: CDCA and GCDCA inhibit the inflammatory response induced by SARS-CoV-2 in THP-1 cells; Table S1: The sequence of qRT-PCR primer; Table S2: Comparison of personal information, clinical characteristics, and immune indicators between the Non-severe and Severe groups of elderly COVID-19 patients; Table S3: The full name of pathways enriched by Reactome analysis.
