# Supplementary material for: Significant Reduction of Chenodeoxycholic Acid and Glycochenodeoxycholic Acid in the Elderly with Severe COVID-19
Source: Biomolecules. 2025 Jun 28;15(7):943. doi: 10.3390/biom15070943 (PMC12292118; doi:10.3390/biom15070943)
Supplement: Supplementary file 1 [file biomolecules-15-00943-s001.zip › biomolecules-3645060-supplementary.pdf]

Supplementary Table S1. The sequence of qRT-PCR primer

Target gene	Forward primer sequence	Reverse primer sequence
IL-1α	AGATGCCTGAGATACCCAAAACC	CCAAGCACACCCAGTAGTCT
IL-1β	AACCTCTTCGAGGCACAAGG	AGATTCGTAGCTGGATGCCG
IL-6	ATGCAATAACCACCCCTGACC	CCATGCTACATTTGCCGAAGAG
IL-8	AAGGTGCAGTTTTGCCAAGGAGT	CGCAGTGTGGTCCACTCTCAATC
TNF-α	CCTCTCTCTAATCAGCCCTCTG	GAGGACCTGGGAGTAGATGAG
CXCL5	GAGAGAGCTGCGTTGCGTTT	TTCAGGGAGGCTACCACTTC
FXR	GCAGCCTGAAGAGTGGTACT	CAACACACAGCTCATCCCCT
TGR5	TCAGCCAGGACACCAGACAT	TGGGCCTTCCTGAGTGTCA
β-Actin	CTCGCCTTTGCCGATCC	GTACTTCAGGGTGAGGATGC

Supplementary Table S2. Comparison of personal information, clinical characteristics, and immune indicators between the Non-severe and Severe groups of elderly COVID-19 patients

	**Non-severe group (n=23)**	**Severe group (n=8)**	**P-value**
**Personal information**			
Sex			
Male	17(73.91%)	8(100.00%)	0.298
Female	6(26.09%)	0(0.00%)	
Age (year)	80(71,86)	81(70,89)	0.707
Underlying comorbidities			
Circulatory System Diseases	16(69.57%)	6(75.00%)	1.000
Endocrine System Diseases	13(56.52%)	5(62.50%)	1.000
Digestive System Diseases	6(26.09%)	1(12.50%)	0.642
Nervous System Diseases	4(17.39%)	1(12.50%)	1.000
Urinary System Diseases	1(4.35%)	4(50.00%)	0.010*
Respiratory System Diseases	1(4.35%)	3(37.50%)	0.043*
Musculoskeletal System	4(17.39%)	1(12.50%)	1.000
Others	1(4.35%)	1(12.50%)	0.456
**Clinical information**			
Time from the onset to sample collection (day)	5(2,10)	7(4,9)	0.674
**Immune-related parameters**			
Leukocyte(10^9/L)	4.56(3.42,6.47)	5.32(3.47,8.55)	0.520
Neutrophils(%)	73.14±16.57	84.60±10.381	0.078
Lymphocytes(%)	18.32±12.81	11.46±9.43	0.177
Monocytes (%)	7.00±5.00	3.30±1.60	0.046*
Neutrophils(10^9/L)	3.59(2.48,4.53)	4.22(3.12,7.80)	0.255
Lymphocytes(10^9/L)	0.85±0.58	0.54±0.39	0.168
Monocytes(10^9)/L)	0.33±0.26	0.21±0.15	0.227
PCT(ng/ml)	0.04(0.01,0.10)	0.09(0.03,0.24)	0.121
CRP(mg/L)	21.20(5.00,85.80)	58.40(30.00,108.500)	0.156
IL-6(pg/ml)	10.58(4.67,24.28)	8.80(4.70,24.70)	0.893

Note: Data of CRP is missing for 6 patients in the Non-severe group and 1 patient in the Severe group; data of IL-6 is missing for 9 patients in the Non-severe group and 3 patients in the Severe group. * means P-value<0.05. Circulatory system diseases included hypertension, coronary heart disease, arrhythmias, peripheral artery disease, stroke. Endocrine system diseases included diabetes, gout, hyperuricemia. Digestive system diseases included

gastroesophageal reflux disease, peptic ulcers. Nervous system diseases included Alzheimer's disease, Parkinson's disease, epilepsy, multiple sclerosis, peripheral neuropathy, stroke, migraines, stun, neurogenic deafness, depression, insomnia and anxiety. Urinary system diseases included chronic kidney disease, kidney stones, urinary tract infections and hyperplasia of prostate. Respiratory system diseases included chronic obstructive pulmonary disease, asthma, interstitial pneumonia and pulmonary nodules. Musculoskeletal system diseases included osteoarthritis, osteoporosis, muscular dystrophy, lumbar disc herniation, paralysis and fractures. Others included pressure sore, cataracts, glaucoma and eczema.

Supplementary Table S3. The full name of pathways enriched by Reactom analysis

ID	The full name of pathway
R-HSA-193368	Synthesis of bile acids and bile salts via 7alpha-hydroxycholesterol
R-HSA-192105	Synthesis of bile acids and bile salts
R-HSA-194068	Bile acid and bile salt metabolism
R-HSA-8957322	Metabolism of steroids
R-HSA-2142816	Synthesis of (16-20)-hydroxyeicosatetraenoic acids (HETE)
R-HSA-70350	Fructose catabolism
R-HSA-71288	Creatine metabolism
R-HSA-1989781	PPARA activates gene expression
R-HSA-400206	Regulation of lipid metabolism by PPARalpha

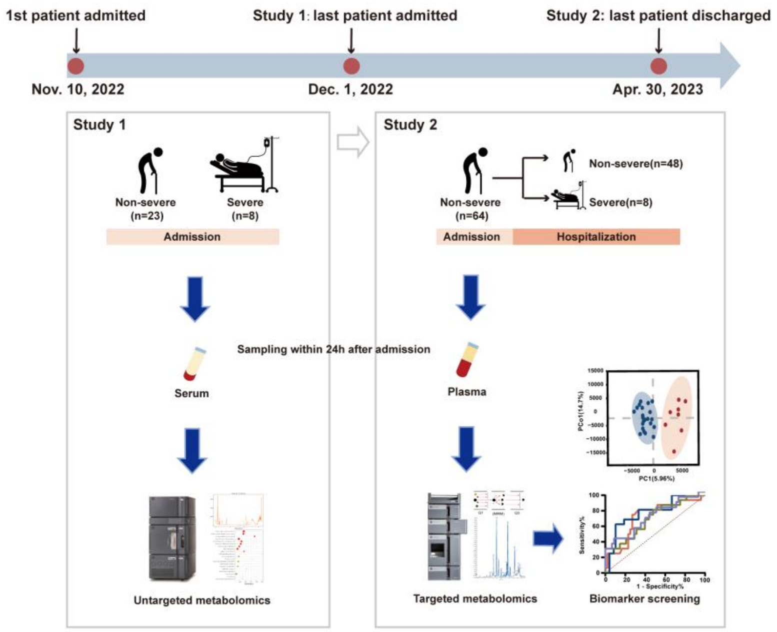

Supplementary Figure S1. Timeline of patient enrollment, sampling and detection process.

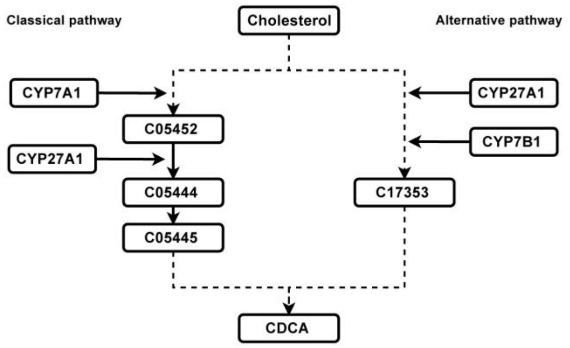

Supplementary Figure S2. Differential metabolites on the primary bile acid biosynthesis pathway.

Note: C05452: 3a,7a-Dihydroxy-5b-cholestane; C05444: 3a,7a,26-Trihydroxy-5beta-cholestane; C05445:3a,7a-Dihydroxy-5b-cholestan-26-al; C17353: 3b,7a-Dihydroxy-5-cholestenoate. The solid line represents direct action, the dashed line represents indirect action.

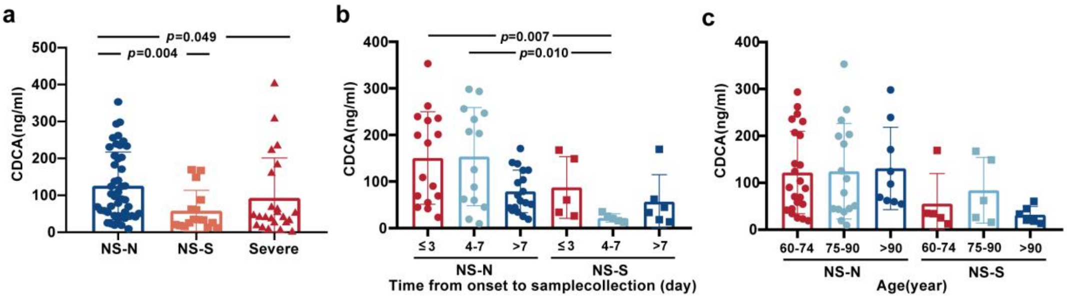

Supplementary Figure S3. The expression of CDCA detected by targeted metabolomics in the peripheral blood of elderly COVID-19 patients at admission. **(a)** Comparison of CDCA levels among NS-N, NS-N and Severe groups. **(b)** Comparison of CDCA levels in NS-N and NS-S at different times from onset to sample collection. **(c)** Comparison of CDCA levels in NS-N and NS-S at different age groups. Data are presented as median and interquartile range. The two-sided P values were examined using Kruskal-Wallis test followed by Dunn's multiple-comparison test for comparison of continuous variables among multiple groups **(a-c)**.

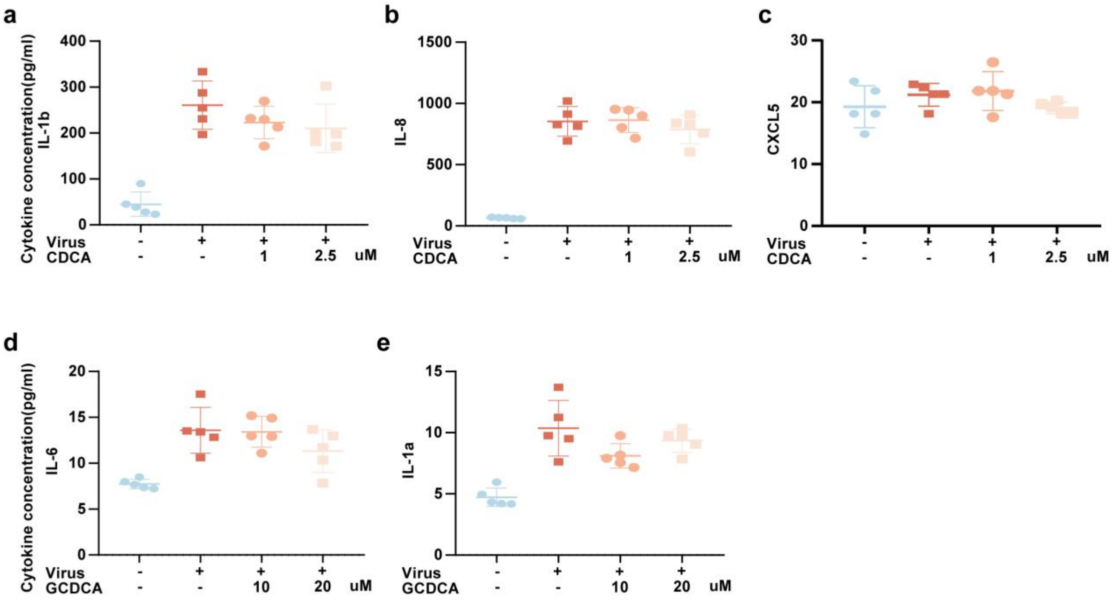

Supplementary Figure S4. CDCA and GCDCA inhibit the inflammatory response induced by SARS-CoV-2 in THP-1 cells. **(a,b)** SARS-CoV-2 stimulated THP-1 cells were treated with 1uM or 2.5uM CDCA for 24 h. The supernatant protein concentrations of IL-1β (**a**) and IL-8 (**b**) were measured by CBA, CXCL5 (**c**) level was measured by ELISA. **(d,e)** SARS-CoV-2 stimulated THP-1 cells were treated with 10uM or 20uM GCDCA for 24 h. The supernatant protein concentrations of IL-6 (**d**) and IL-1α (**e**) were measured by CBA.
